# Zonular instability-associated morphologic features in eyes with primary angle closure disease using the swept-source anterior segment – optical coherence tomography system

**DOI:** 10.1186/s12886-024-03462-1

**Published:** 2024-04-30

**Authors:** Xue-Ting Pei, Shu-Hua Wang, Guo-Ping Qing, Xiao-Wei Yu, Yan Shi, Wen-Li Yang, Ning-Li Wang, Zhi-Gang Fan

**Affiliations:** Beijing Ophthalmology and Visual Science Key Laboratory, Beijing Tongren Eye Center, Beijing Tongren Hospital, Capital Medical University, Beijing, 100730 China

**Keywords:** PACD, Zonular instability, CASIA 2 AS-OCT, Lens anterior curvature, Lens anterior part thickness

## Abstract

**Background:**

This study aims to investigate the morphologic features of the crystalline lens in Primary Angle Closure Disease (PACD) patients with zonular instability during cataract surgery using the swept-source CASIA 2 Anterior Segment-Optical Coherence Tomography (AS-OCT) system.

**Methods:**

A total of 398 eyes (125 PACD eyes with zonular instability, 133 PACD eyes with zonular stability, and 140 cataract patient controls) of 398 patients who underwent cataract surgery combined or not glaucoma surgery between January 2021 and January 2023 were enrolled. The crystalline lens parameters were measured by CASIA2 AS-OCT. Then, logistic regression was performed to evaluate the risk factors associated with zonular instability.

**Results:**

The results revealed that PACD eyes had a more anterior lens equator position, a steeper anterior curvature of lens, shorter Axial Length (AL), shallower Anterior Chamber Distance (ACD), higher Lens Vault (LV) and thicker Lens Thickness (LT), when compared to eyes in the cataract control group. Furthermore, PACD eyes in the zonular instability group had steeper front R, front Rs and Front Rf, flatter back Rf, thicker lens anterior part thickness, higher lens anterior-to-posterior part thickness ratios, shallower ACD, and greater LV, when compared to PACD eyes with zonular stability. The logistic regression analysis, which was adjusted for age and gender, revealed that zonular instability was positively correlated with anterior part thickness, lens anterior-to-posterior part thickness ratio, and LV, but was negatively correlated with lens anterior radius and ACD.

**Conclusion:**

Steeper anterior curvature, increased lens anterior part thickness, higher anterior-to-posterior part thickness ratio, shallower ACD, and greater LV are the anatomic features of PACD eyes associated with zonular instability.

## Background

Primary angle-closure glaucoma (PACG), the leading cause of irreversible visual impairment and blindness, is the most severe form of primary angle closure disease (PACD) with the highest incidence in the Asian population [1, 2]. PACD is characterized by appositional or synechial closure of the anterior chamber angle by the peripheral iris, and can be classified as primary angle closure suspect (PACS, defined as narrow angles predisposed to angle closure), primary angle closure (PAC, defined as occludable angle and trabecular obstruction), and PACG with evidence of glaucomatous optic neuropathy [3, 4].

Primary phacoemulsification (phaco) in combination with intraocular lens (IOL) implantation has been regarded as the first-line therapy for PACD [5, 6]. Multicenter clinical trials have demonstrated that cataract extraction significantly increases the anterior chamber angle, and improves the anterior segment configuration [5]. However, lens zonular instability and weakness have been frequently observed in PACD eyes during cataract surgery, and when lens zonular instability and weakness occurs, the use of capsular tension rings (CTR) or occasionally sclera-fixated IOL (SF-IOL) is recommended [7]. Hence, the prediction of zonular instability in clinic is important for surgical scheduling.

The preoperative signs of lens subluxation caused by partial zonular dehiscence include iridodonesis, phacodonesis, visibility of the lens equator, and asymmetric anterior chamber depth (ACD) with the appearance of uneven depth of the anterior chamber. Lens subluxation secondary angle-closure glaucoma can be easily misdiagnosed as acute PACG, which is featured by a shallower ACD [8, 9]. However, since PACD eyes with an originally shallow ACD in both eyes may not have the feature of malposition of the lens, it remains difficult to discriminate whether there is zonular instability in PACD eyes before surgery using a slit lamp, ocular B-mode ultrasound, gonioscopy, or UBM.

Kwon et al. used first generation anterior segment-optical coherence tomography (AS-OCT) to identify the zonular instability in the eyes after an acute angle closure attack, and showed eyes with zonular instability exhibit less hyperopic spherical equivalent (SE), longer axial length (AL), shallower ACD, and higher lens vault (LV) values, when compared to eyes with zonular stability [7]. Recently, swept-source AS-OCT (CASIA 2, Tomey Corporation, Nagoya, Japan) has been used to distinguish PACGs. The AS-OCT device, with a wide scanning range (16 mm), can simultaneously capture the anterior and posterior lens surface, and calculate the lens parameters, including the curvature, decentration and tilt of the lens. The present study aimed to explore the lens morphologic features of PACD eyes with zonular instability during cataract surgery using the CASIA 2 AS-OCT system.

## Methods

### Subjects

The present retrospective study was approved by the institutional review board of Beijing Tongren Hospital (approval number: TRECKY2020-056), and was conducted at the Eye Center of Beijing Tongren Hospital, in accordance with the tenets of the Declaration of Helsinki. The present study consecutively enrolled 125 PACD eyes with zonular instability, 133 PACD eyes with zonular stability, and 140 cataract eyes of 398 patients, who underwent cataract surgery between January 2021 and January 2023, at the Eye Center of Beijing Tongren Hospital. A written informed consent was obtained from all participants, and all participants were informed about the study prior to inclusion.

### Diagnosis of PACD with zonular instability or zonular stability and cataract

The medical records of all patients diagnosed with PACD (PACS, PAC, or PACG), age-related cataract (including nuclear cataract, cortical cataract, and subcapsular cataract) patients, and patients who underwent phaco and IOL implantation surgery in combination with or not glaucoma surgery were reviewed and recorded. PACS was defined as an eye with a pigmented trabecular meshwork invisible for 180° or greater under static gonioscopy, but without peripheral anterior synechiae, IOP, or glaucomatous optic neuropathy. PAC was defined as an eye with the presence of peripheral anterior synechiae or elevated IOP, but without glaucomatous optic neuropathy. PACG was defined as an eye with PAC and glaucomatous optic neuropathy. These patients were categorized into two groups, based on their zonular stability status during the cataract surgery: zonular stability group and zonular instability group.

Inclusion criteria for the zonular instability group: (1) no signs of lens subluxation before surgery, such as asymmetric ACD with the appearance of uneven depth of the anterior chamber, visibility of the lens equator, iridodonesis, and phacodonesis; (2) abnormal lens position was detected or not by preoperative UBM examination; (3) zonular weakness detected by experienced surgeons during cataract surgery, according to the significant intraoperative signs, including peripheral light transmission, anterior capsular wrinkles and poor follow-up of the anterior capsular membrane during continuous circular capsulorhexis, instability of the capsule during phaco and cortex irrigation, non-circular form of the anterior capsule mouth after irrigation.

Inclusion criteria for the zonular stability group: (1) no signs of lens subluxation, such as asymmetric ACD with the appearance of uneven depth of the anterior chamber, visibility of the lens equator, iridodonesis, and phacodonesis; (2) abnormal lens position was not detected by preoperative UBM; (3) the zonule and lens capsule were stable during cataract surgery.

Patients in the zonular stability group underwent micro-incision phacoemulsifiction (2.2 mm) and in-the-bag single-piece IOL implantation, while patients in the zonular instability group (including eyes with zonular weakness) mostly underwent CTR insertion and/or occasionally SF-IOL. All of the surgeries were performed by the four highly experienced surgeons (Zhigang Fan, Guoping Qing, Shuhua Wang, and Xueting Pei).

The age- and gender-matched control patients included age-related cataract patients who underwent phacoemulsification and IOL implantation.

Inclusion criteria for cataract patients: patients with age-related cataract (including nuclear cataract, cortical cataract, and subcapsular cataract); patients > 50 years old; patients with an AL of < 24 mm; patients without a history of intraocular surgery, laser treatment, ocular diseases and ocular trauma, and corticoid use; patients with stable zonule and lens capsular during cataract surgery.

Patients who underwent incisional surgery and had a history of eye diseases (such as uveitis and trauma), and intraocular surgery, or used pilocarpine were excluded. For patients who met the criteria for both eyes, the eye with the more severe disease condition was included for the analysis.

### Ophthalmic examination and AS-OCT imaging

All patients underwent ophthalmic examinations, including slit-lamp biomicroscopy, best-corrected visual acuity measurements, gonioscopy, funduscopic examination, visual field test using the Humphrey Visual Field Analyzer II, intraocular pressure measurement by Goldmann applanation tonometry, stereoscopic optic disc photography, and UBM. The lens opacity was graded according to the Lens Opacities Classification System III standards: nuclear opalescence (NO; score, 1–6), nuclear color (NC; score, 1–6), cortical (C; score, 1.00–5.19), and posterior subcapsular cataract (P; score, 1.00–5.19).

**Fig. 1:**
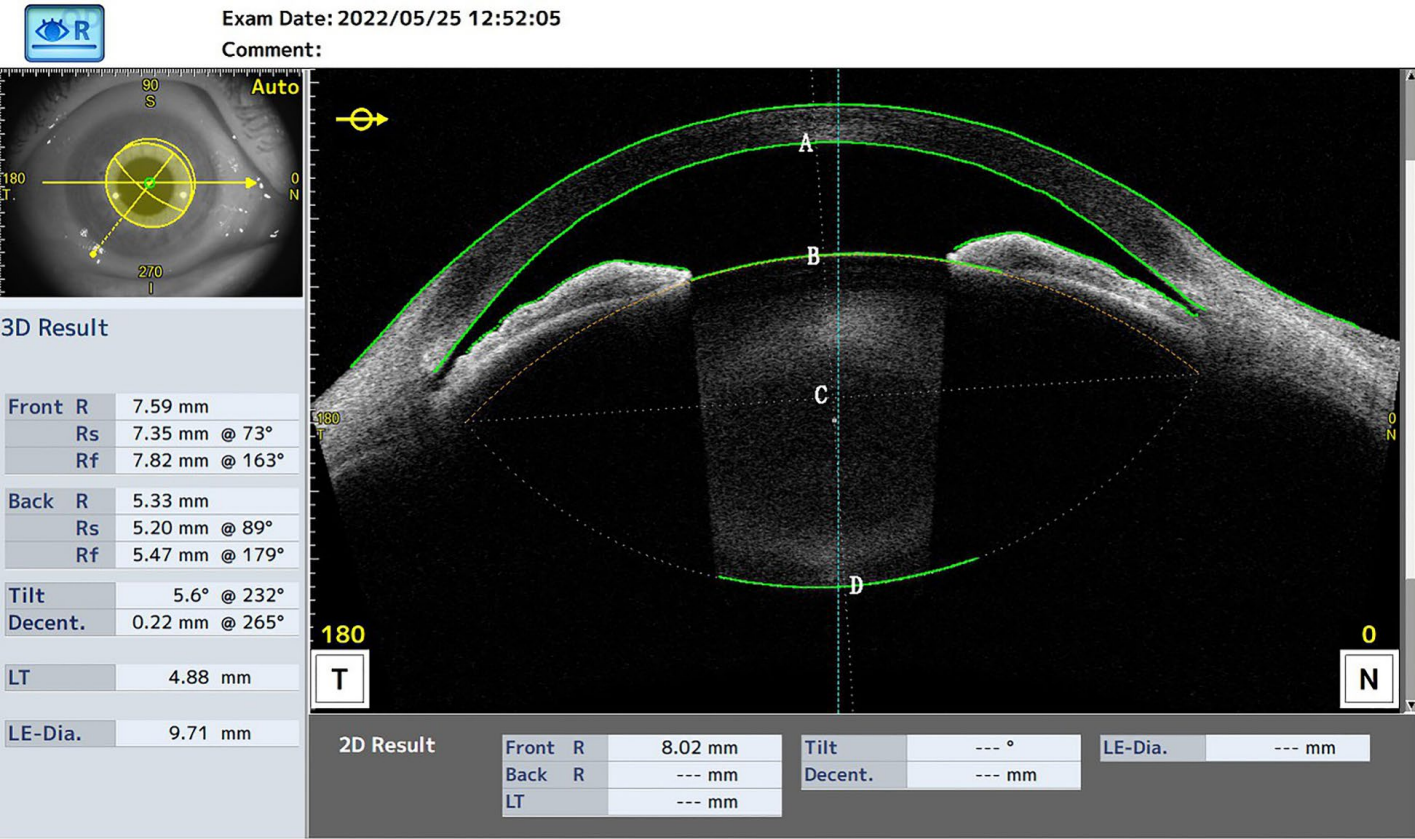
The Casia 2 image for the anterior segment. **A** Intersection of the corneal endothelium and vertex normal. **B** Intersection of the lens anterior surface and vertex normal. **C** Intersection of the lens center and vertex normal. **D** Intersection of the lens posterior surface and vertex normal. Lens equator depth (Line AC). Lens anterior part thickness (Line BC). Lens posterior part thickness (Line CD)

All patients underwent AS-OCT (CASIA 2, Tomey Corporation, Nagoya, Japan) imaging using a swept source laser at a wavelength of 1,310 nm (frequency, 0.3 s). The CASIA2 AS-OCT system produced 16 images from 16 different two-dimensional angles, and obtained the outline of the lens with the anterior and posterior curvature of lens (Fig. 1). The three-dimensional crystalline lens morphology was constructed from the 16 different images, and the following lens parameters were obtained: front Rs and back Rs (the anterior and posterior radius of the steep curvature of the lens, respectively), front Rf and back Rf (the anterior and posterior radius of the flat curvature of the lens, respectively), front R and back R (mean value of the anterior and posterior Rs and Rf of the lens, respectively), lens thickness (LT, along the vertex normal), and lens vault (LV, vertical distance from the anterior lens surface to the horizontal line that connects the two scleral spurs). The lens equator depth (AC in Fig. 1) was determined using the distance from the corneal endothelium to the lens equator line. The lens anterior part thickness (BC in Fig. 1) was determined using the distance from the anterior lens surface to the lens center along the vertex normal. The lens posterior part thickness (CD in Fig. 1) was determined using the distance from the lens center to the posterior lens surface along the vertex normal. The lens anterior-to-posterior part thickness ratio was calculated as BC / CD. Decentration was defined as the vertical distance from the lens center to the vertex normal. The tilt was determined using the angle of the lens axis against the vertex normal.

### Statistical analysis

SPSS version 20.0 (SPSS Inc., Chicago, Illinois, USA) was used for the statistical analysis. Numerical variables were presented as mean ± standard deviation (SD). The differences among the three groups were compared by analysis of variance (ANOVA) and Kruskal–Wallis test. For categorical variables, the difference was compared using Chi-square test. Univariate logistic regression was conducted to evaluate the relationships between the lens parameters and zonular instability. The predictors of zonular instability were determined by performing a logistic regression analysis adjusted by age and gender. A *P*-value of < 0.05 was considered statistically significant.

## Results

A total of 398 eyes of 398 subjects were enrolled for the present study: 125 PACD eyes with zonular instability, 133 PACD eyes with zonular stability, and 140 control cataract eyes. Table 1 summarizes the demographic and basic clinical information of all patients. Age and gender were not significantly different among the three groups (all, *P* > 0.05). However, there were significant differences in AL (*P* = 0.000), NO (*P* = 0.009), NC (*P* = 0.009), C (*P* = 0.006), and P (*P* = 0.004). Furthermore, the PACD groups had shorter AL, and the zonular instability group had the lowest NO, NC, C and P scores.

**Table 1:** Clinical characteristics of participants

	**PACD patients**		**Cataract patients**	
**Variables**	**Zonular instability (*n* = 125)**	**Zonular stability (*n* = 133)**	**Control** **(*n* = 140)**	***P*-value**
Gender (Male:Female)	46:79	39:94	56:84	0.429
Age	66.76 ± 6.379	64.10 ± 8.375	65.84 ± 8.510	0.554
AL master	22.56 ± 0.76	22.30 ± 0.54	23.47 ± 0.66	0.000
NO	1.1 ± 1.0	1.6 ± 1.0	1.7 ± 1.0	0.009
NC	1.1 ± 1.0	1.6 ± 1.0	1.7 ± 1.0	0.009
C	1.4 ± 0.9	2.0 ± 0.9	2.3 ± 1.1	0.006
P	1.0 ± 0.8	1.8 ± 0.7	2.0 ± 1.2	0.004

The differences were determined by ANOVA test and Kruskal–Wallis test

*PACD *Primary angle closure disease, *AL *Axial length, *NO *Nulear opacification, *NC *Nuclear cataract, *C *Cortical cataract, *P *Subcapsular cataract

Table 2 summarizes the lens biometric parameters measured using the CASIA 2 AS-OCT system. There were significant differences in ACD (*P* < 0.001), LV (*P* < 0.001), front R (*P* < 0.001), front Rs (*P* < 0.001), front Rf (*P* < 0.001), LT (*P* < 0.001), lens equator depth (*P* < 0.001), lens anterior part thickness (*P* < 0.001), lens posterior part thickness (*P* = 0.004), and the anterior-to-posterior part thickness ratio of the lens (*P* = 0.003) among the three groups. However, there were no significant differences in lens tilt (*P* = 0.538) and decentration (*P* = 0.110) among the three groups.

**Table 2:** Lens biometric parameters for the three groups measured by the Casia 2 AS-OCT system

	**PACD patients** ***n* = 258**		**Cataract patients**		
**Variables**	**Zonular instability** ***n* = 125**	**Zonular stability** ***n* = 133**	**Control** ***n* = 140**	***F*-value**	***P*-value**
ACD	1.65 ± 0.22	1.92 ± 0.25	2.51 ± 0.34	43.479	0.000
LV	1.29 ± 0.25	1.01 ± 0.27	0.43 ± 0.46	20.322	0.000
ACW	11.30 ± 0.27	11.39 ± 0.34	11.59 ± 0.57	2.633	0.090
Front R	7.64 ± 0.42	8.25 ± 0.64	9.47 ± 0.92	42.846	0.000
Front Rs, mm	7.36 ± 0.38	7.83 ± 0.54	9.14 ± 0.88	38.443	0.000
Front Rs Axis, °	74.73 ± 64.73	79.52 ± 49.79	73.36 ± 25.73	0.096	0.909
Front Rf, mm	8.13 ± 0.61	8.75 ± 0.88	9.86 ± 0.95	22.934	0.000
Front Rf Axis, °	106.13 ± 51.81	118.86 ± 57.94	128.85 ± 68.67	0.207	0.650
Back R, mm	5.76 ± 0.82	5.53 ± 0.32	5.72 ± 0.35	2.517	0.109
Back Rs, mm	5.40 ± 0.60	5.29 ± 0.43	5.47 ± 0.41	1.178	0.315
Back Rs Axis, °	78.20 ± 46.96	97.09 ± 55.20	76.37 ± 48.58	1.189	0.312
Back Rf, mm	6.06 ± 0.67	5.75 ± 0.45	5.98 ± 0.38	2.536	0.088
Back Rf Axis, °	103.20 ± 60.23	85.88 ± 46.87	94.20 ± 55.43	0.442	0.645
Lens thickness	5.20 ± 0.34	5.12 ± 0.26	4.55 ± 0.29	27.462	0.000
Lens diameter	9.98 ± 0.53	10.08 ± 0.36	10.20 ± 0.34	0.804	0.453
Lens tilt	6.08 ± 1.62	5.45 ± 1.39	6.04 ± 2.12	0.627	0.538
Tilt axis, °	201.07 ± 78.99	250.12 ± 75.07	262.30 ± 86.70	1.917	0.158
Lens decentration	0.20 ± 0.08	0.15 ± 0.06	0.19 ± 0.08	2. 290	0.110
Decentration axis, °	199.53 ± 69.605	193.35 ± 98.148	215.45 ± 117.071	0.331	0.720
Lens equator depth	3.35 ± 0.38	3.54 ± 0.22	4.13 ± 0.23	43.826	0.000
Lens anterior part thickness	1.96 ± 0.27	1.72 ± 0.21	1.53 ± 0.16	18.546	0.000
Lens posterior part thickness	3.16 ± 0.47	3.35 ± 0.31	2.98 ± 0.25	5.397	0.007
Anterior part thickness: Posterior part thickness	0.66 ± 0.15	0.51 ± 0.10	0.51 ± 0.06	6.464	0.003

*ACD* Anterior chamber depth, *LV* Lens vault, *ACW* Anterior chamber wide, *Front R* Mean value of lens front radius, *front Rs* front radius of the steep curvature of the lens, *front Rf* lens front radius of the flat curvature of the lens, *Back R* mean value of lens back radius, *Back Rs* back radius of the steep curvature of the lens, *Back Rf* back radius of the flat curvature of the lens, *LT* Lens thickness

Compared to the control group, the PACD groups had shorter ACD and lens equator depth, greater LV, LT, lens anterior part thickness, and anterior-to-posterior part thickness ratios, and steeper front R, front Rs and front Rf (all, *P* < 0.05; Table 3).

**Table 3:** Statistical significance between two groups

Variables	Zonular instability *vs.* Zonular stability	Zonular instability *vs.* Controls	Zonular stability *vs.* Controls	Combined PACD vs. Control
Age	0.802	0.702	0.906	0.853
AL	0.085	0.000	0.000	0.000
ACD	0.017	0.000	0.000	0.000
LV	0.006	0.000	0.000	0.000
ACW	0.555	0.101	0.328	0.030
Front R	0.005	0.000	0.001	0.000
Front Rs, mm	0.033	0.000	0.000	0.000
Front Rs axis, °	0.767	0.919	0.675	0.747
Front Rf, mm	0.030	0.000	0.000	0.000
Front Rf axis, °	0.850	0.564	0.17	0.509
Back R, mm	0.085	0.927	0.119	0.739
Back Rs, mm	0.269	0.690	0.147	0.279
Back Rs axis, °	0.184	0.992	0.196	0.447
Back Rf, mm	0.042	0.759	0.087	0.405
Back Rf axis, °	0.386	0.460	0.920	0.727
Lens thickness	0.352	0.000	0.000	0.000
Lens diameter	0.441	0.650	0.217	0.318
Lens tilt	0.325	0.959	0.359	0.605
Tilt axis,	0.052	0.059	0.815	0.193
Lens decentration	0.061	0.743	0.109	0.447
Decentration axis,	0.957	0.508	0.460	0.416
Lens equator depth	0.052	0.000	0.000	0.000
Lens anterior part thickness	0.001	0.000	0.065	0.000
Lens posterior part thickness	0.088	0.121	0.002	0.008
Anterior part thickness: Posterior part thickness	0.000	0.003	0.865	0.092

Compared to the zonular stability group, eyes in the zonular instability group had shallower ACDs (*P* = 0.017), greater LVs (*P* = 0.006), steeper front R (*P* = 0.005), front Rs (*P* = 0.033) and front Rf (*P* = 0.030), flatter back Rf (*P* = 0.042), thicker lens anterior part thickness (*P* = 0.001), and higher anterior-to-posterior part thickness ratios (*P* = 0.000) (Table 3).

Univariate logistic regression, and age- and gender-adjusted logistic regression analysis were performed to identify the baseline parameters for predicting PACD. The logistic regression analysis revealed that shallower ACD, shorter lens equator depth, greater LV, LT and lens anterior part thickness, and steeper front R, front Rs and front Rf were associated with higher risk for PACD (Table 4).

**Table 4:** Univariate logistic regression, and age- and gender-adjusted logistic regression analysis to assess the baseline parameters for predicting PACD

**Baseline** **Parameters**	**Interval**	***B*-value**	**OR (95% CI)**	***P*-value**	***B*-value**	**OR (95% CI)**	***P*-value**
					**Adjusted for age and gender**		
ACD	0.1 mm	-0.536	0.585 (0.448–0.764)	0.000	-0.963	0.382(0.226–0.646)	0.000
LV	0.1 mm	0.812	2.253(1.492–3.402)	0.000	1.018	2.769(1.536–4.993)	0.001
Front R	1.0 mm	-2.566	0.077(0.022–0.263)	0.000	-2.532	0.079(0.022–0.286)	0.000
Front Rs	1.0 mm	-2.745	0.0664(0.017–0.241)	0.000	-2.877	0.056(0.012–0.257)	0.000
Front Rf	1.0 mm	-1.857	0.156(0.060–0.405)	0.000	-1.746	0.174(0.066–0.460)	0.000
Lens equator depth	0.1 mm	-1.134	0.322(0.170–0.610)	0.002	-1.358	0.257(0.107–0.615)	0.006
Lens anterior part thickness	0.1 mm	0.610	1.840(1.252–2.705)	0.002	0.512	1.669(1.151–2.422)	0.007
Lens thickness	0.1 mm	0.937	2.552(1.551–4.199)	0.001	1.042	2.835(1.468–5.475)	0.002

*Abbreviation*: *ACD* Anterior chamber depth, *LV* Lens vault, *Front R* mean value of lens front radius, *Front Rs* front radius of the steep curvature of the lens, *Front Rf* Lens front radius of the flat curvature of the lens

Univariate logistic regression, and age- and gender-adjusted logistic regression analysis were also performed to identify the baseline parameters for predicting the zonular instability. In the age- and gender-adjusted logistic regression model, ACD, LV, front R, lens anterior part thickness, and the anterior-to-posterior part thickness ratio were significantly associated with zonular instability (all, *P* < 0.05). The 0.1-mm decrease in ACD was associated with the increased risk of zonular instability by 1.545 folds. The 1-mm decrease in front R was associated with the increased risk of zonular instability by 8.696 folds. The 0.1-mm increase in LV and lens anterior part thickness, and the 0.1 increase in anterior-to-posterior part thickness ratio were associated with the increased risk of zonular instability by 1.713, 1.572 and 2.525 folds, respectively (Table 5).

**Table 5:** Univariate logistic regression, and age- and gender-adjusted logistic regression analysis to assess the baseline parameters for predicting zonular instability

Baseline Parameters	Interval (increase)	*B*-value	OR (95% CI)	*P*-value	*B*-value	OR (95% CI)	*P*-value
					**Adjusted for age and gender**		
ACD	0.1 mm	-0.449	0.638(0.453–0.900)	0.010	-0.435	0.647(0.450–0.931)	0.019
LV	0.1 mm	0.476	1.609(1.169–2.215)	0.004	0.538	1.713(1.148–2.554)	0.008
Front R	1.0 mm	-2.277	0.103(0.021–0.506)	0.005	-2.159	0.115(0.023–0.575)	0.008
Front Rs	1.0 mm	-2.591	0.075(0.010–0.588)	0.014	-2.316	0.099(0.012–0.814)	0.031
Front Rf	1.0 mm	-1.110	0.330(0.118–0.922)	0.034	-1.102	0.332(0.116–0.955)	0.041
Lens anterior part thickness	0.1 mm	0.427	1.533 (1.155–2.035)	0.003	0.453	1.572 (1.1532.144)	0.004
Anterior part thickness: Posterior part thickness	0.01	0.757	2.132(1.253–3.626)	0.005	0.926	2.525(1.316–4.847)	0.005

*ACD* Anterior chamber depth, *LV* Lens vault, *ACW* Aanterior chamber wide, *Front R* mean value of lens front radius, *Front Rs* front radius of the steep curvature of the lens, *Front Rf* lens front radius of the flat curvature of the lens

## Discussion

It has been reported that the crystalline lens (e.g. lens thickness and position) contributes to the development of PACG [10–16]. Recently, zonular laxity has frequently occurred during cataract surgery in PACG eyes [9, 17]. However, it remains difficult to identify PACD eyes with zonular instability during cataract surgery through the morphologic features of the crystalline lens in the preoperative examination. To our knowledge, the present study is the first to evaluate the lens morphologic features of PACD eyes with zonular instability during cataract surgery using the CASIA2 AS-OCT system.

The present cross-sectional study revealed that PACD eyes had a more anterior lens equator position, steeper anterior curvature lens, shorter ALs, shallower ACDs, higher LVs, and thicker LTs, when compared to control cataract eyes. More importantly, PACD eyes with zonular instability had steeper front R, flatter back Rf, thicker lens anterior part thickness, higher anterior-to-posterior part LT ratios, shallower ACDs, and greater LVs, when compared to PACD eyes with zonular stability. Furthermore, zonular instability was positively correlated with anterior part thickness, lens anterior-to-posterior part thickness ratio and LV, but negatively correlated with ACD.

Limited by technology, the anterior and posterior surface of the lens cannot be quantitatively displayed well by first-generation AS-OCT and UBM. A previous study reported that compared with normal eyes, PACD eyes exhibited greater iris-lens contact distance using UBM [18], and a more anterior lens position (defined as ACD + 1/2LT) using A-mode applanation ultrasonography [19]. However, Sihota et al*.* reported no differences in lens position (defined as [ACD + 1/2LT] / AL) for angle closure eyes and open angle eyes [15]. The present study used the lens equator depth as a parameter to evaluate the lens position. PACD eyes had a shorter lens equator depth, when compared to control cataract eyes. However, the lens equator depth for PACD eyes with zonular instability was not significantly different, when compared to PACD eyes with zonular stability.

Sun et al*.* reported that PACD eyes exhibited a steeper anterior curvature, when compared to normal eyes (8.07 ± 0.58 in PACS and 7.81 ± 0.51 in PAC/PACG eyes) [20]. Using the Casia 2 OCT, Liu et al*.* reported an average front anterior curvature of 9.73 ± 1.36 in 1,097 cataract patients [21], which is similar to the report of the present study for cataract control eyes (9.44 ± 0.94). Furthermore, compared to cataract control eyes, the front anterior curvature was steeper in PACD eyes, and PACD eyes with zonular instability (7.64 ± 0.42) had a much steeper anterior curvature, when compared to PACD eyes with zonular stability (8.25 ± 0.64).

In addition, the present study revealed that the lens anterior part thickness and anterior-to-posterior part thickness ratio were highest in PACD eyes with zonular instability, and were more sensitive for predicting the zonular weakness, when compared to the front anterior curvature. Similar to the present results, a previous study revealed that the entire LT was thicker in PACD eyes, when compared to control cataract eyes [16]. Furthermore, the present study revealed a anterior-to-posterior lens part thickness ratio of 0.51 for both PACD eyes with zonular stability and control cataract eyes, and that this increased to 0.66 ± 0.15 in PACD eyes with zonular instability. These findings suggest that the anterior-to-posterior lens part thickness ratio is sensitive for predicting the zonular instability.

Overall, lens morphologic changes, including the steep front anterior curvature and increased anterior part LT identified by the CASIA2 AS-OCT system, might contribute to the increase in LV and decrease in ACD in PACD patients, especially in PACD patients with zonular instability. Thus, these lens morphologic changes, which partially relies on the tension of the zonula, might be the anatomic characteristics of PACD eyes with zonular instability.

The present study had no significant difference in lens decentration and lens tilt among the zonular instability, zonular stability, and control groups. Different from the present study, Sun et al*.* reported that compared to normal eyes, PACD eyes exhibited greater lens decentration and tilt, which was presumed to be correlated with the zonular weakness [20]. However, in that study, the PACD patients were not sub-categorized according to zonular stability, and the differences in lens decentration and tilt in PACG eyes and controls were minimal. In addition, Liu et al*.* reported that the cataract lens exhibited an inferotemporal tilt of 5.16 degrees and a temporal decentration of 0.22 mm [21].

The present study had some limitations, which included the relatively small sample size and the systematic errors in biometric measurements. Since the present study was retrospective in nature, merely the correlation between various factors with zonular weakness in PACD was observed, while the underlying causal relationship could not be clarified. In future studies, a cohort study should be performed to identify the relevant indicators for zonular instability, and clarify the mechanism of factors underlying lens zonular weakness in PACD.

## Conclusion

In summary, the present study revealed that PACD eyes with zonular instability exhibit steeper anterior curvatures, thicker anterior part thickness, and higher anterior-to-posterior part thickness ratios, with normal decentration and tilt of the lens. Furthermore, the parameters obtained from the CASIA 2 AS-OCT system, which include steeper front R, thicker anterior part thickness, higher anterior-to-posterior thickness ratio, shallower ACD, and greater LV, are useful for predicting the zonular instability in PACD eyes.

## Data Availability

The datasets generated and analysed during the current study are not publicly available due to limitations of ethical approval involving the patient data and anonymity but are available from the corresponding author on reasonable request.
